# Intestinal resection rates in Crohn’s disease decline across two different epidemiological areas: a consistent observation not merely due to introduction of anti-TNFα

**DOI:** 10.1136/gutjnl-2019-319321

**Published:** 2019-08-14

**Authors:** Evelien M J Beelen, C Janneke van der Woude, Annemarie C de Vries

**Affiliations:** Department of Gastroenterology and Hepatology, Erasmus University Medical Center, Rotterdam, The Netherlands

**Keywords:** Crohn’s disease, surgical resection, intestinal resection, time trends, anti-TNF

With great interest we have read the manuscript written by Murthy *et al*, published in *Gut* in June 2019, on the influence of anti-tumour necrosis factor alpha (TNFα) therapy introduction on the rate of hospitalisation and intestinal resection rates in IBD.^1^ Despite the difference in the source of data between both studies (Canada: health administrative data and the Netherlands: nationwide pathology database), a declining rate of intestinal resections in Crohn’s disease has been confirmed in Canada, at equal rates as the decline that has been observed in the Netherlands.^2^

The authors used an advanced statistical method to analyse the impact of anti-TNFα introduction on (among other end points) the rate of intestinal resection. However, in our opinion, the hypothesised direct relationship between introduction of anti-TNFα and a decline in intestinal resection rate is vastly oversimplified for two important reasons. First, several other factors that have influenced both (early) diagnosis and management of Crohn’s disease should be taken into account. Important changes over the past decades include improved access to endoscopy, less complications at diagnosis and development of strict and non-invasive monitoring strategies. In their original hypothesis, the authors also state the expectation that a similar linear decline during the years before introduction of infliximab would continue during the following years, in the absence of infliximab introduction. In our opinion, the observed decline before introduction of anti-TNFα rather confirms that other factors (as mentioned above) influence time trends of intestinal resection in Crohn’s disease. Second, the effect of anti-TNFα introduction on the progression of Crohn’s disease should preferably be measured on an individual patient level during long-term follow-up. In this publication, intestinal resection rates are only published on an epidemiological and economic level. Data that are essential to translate the epidemiological trends to clinical practice are the timing of anti-TNFα therapy after Crohn’s disease diagnosis (ie, possibly only early medical intervention will impact the risk of intestinal resection), and the individual risk of intestinal re-resection (a marker of long-term prognosis after a ‘reset’ or ‘new onset’ Crohn’s disease after resection).

The conclusion of the authors that the use of infliximab in Crohn’s disease may be misguided as an explanation for the gradually declining rate of intestinal resections seems ingrained by the one factor-effect hypothesis described above. We would rather state more positively that among two different epidemiological areas, the intestinal resection rate in Crohn’s disease is declining, probably as a marker of improved prognosis, attributed to the improvement of care to patients with Crohn’s disease in various ways.
